# Development of a Vocabulary Inventory for English-Mandarin Dual-Language-Learning Infants and Toddlers

**DOI:** 10.3390/bs16071143

**Published:** 2026-07-08

**Authors:** Kristy H. Lai, Huanhuan Shi, Yueting Pan, Catherine S. Tamis-LeMonda

**Affiliations:** Department of Applied Psychology, New York University, 246 Greene St, New York, NY 10003, USA; kristy.lai@nyu.edu (K.H.L.); hs3035@nyu.edu (H.S.); selinapan@link.cuhk.edu.hk (Y.P.)

**Keywords:** bilingualism, infant/toddler vocabulary, dual language learners, language assessment, Communicative Development Inventories (CDI), English, Mandarin

## Abstract

The science of early bilingual language development requires accurate documentation of the words that infants/toddlers acquire in each language to address questions about language transfer, mutual exclusivity, conceptual vocabulary growth, etc. We developed the Dual Language Learners English–Mandarin (DLL-EM) inventories to measure the vocabularies of English–Mandarin infants/toddlers. The inventories expand on American English and Beijing Mandarin MacArthur–Bates Communicative Development Inventories (CDIs) with full translation equivalents and Mandarin dialect options, while retaining original words on CDI forms. We quantified the psychometric properties of the DLL-EM (Study 1) using Item Response Theory based on American English and Beijing Mandarin CDI data from Wordbank and Web-CDI (*n* = 1880, Level 1; *n* = 7536, Level 2). The DLL-EM demonstrated near-perfect correlations with estimated English and Mandarin CDI scores. We interviewed a small group of mothers to characterize the composition of their toddlers’ vocabularies in English and Mandarin (Study 2, *N* = 30). The DLL-EM required ~15 min to administer, yielded large differences among toddlers in English and Mandarin vocabularies, and provided insights into toddlers’ total and conceptual vocabularies that diverged from estimates yielded by nonmatching original CDI forms. The DLL-EM provides a valid, culturally responsive tool for assessing vocabularies of English–Mandarin dual language learners, which is essential for researchers, educators, and clinicians.

## 1. Introduction

### 1.1. Overview

Over the course of the second year, children grow from averaging just a few words to averaging ~300 words (Fenson et al., 2007; Frank et al., 2021; Schneider et al., 2015). Despite impressive vocabulary growth in children as a group, early-emerging individual differences are striking and stable over time. Early vocabulary predicts language skills in preschool (Duff et al., 2015; Hirsh-Pasek et al., 2015; Masek et al., 2021) and elementary school (e.g., Bates et al., 1996; Bleses et al., 2016; Frank et al., 2021) and continues to be the single best predictor of school performance years later (Dale et al., 2023). Thus, an accurate assessment of early vocabulary is essential for researchers, educators, and clinicians.

The widely used, cost-effective MacArthur-Bates Communicative Development Inventories (MB-CDI)—which ask caregivers to report on the vocabularies of their 8- to 30-month-olds—show strong validity and reliability (Fenson et al., 1994, 2007). The MB-CDI forms were originally created and normed on monolingual American English-learning children from the United States (Fenson et al., 2007) and have been adapted to 100+ languages, including Mandarin (Tardif et al., 2009; Hao et al., 2008). These adaptations, commonly referred to as CDIs, attend closely to the cultural and linguistic appropriateness for monolingual children (Marchman & Dale, 2023). However, the use of two original inventories normed on monolingual children may not be appropriate for assessing the vocabularies of dual language learners (DLLs) due to minimal overlap in concepts (translation equivalents) across language forms (Weisleder et al., 2024). Valid inventories with full translation equivalents are critical to accurately document key features of vocabulary growth in children acquiring more than one language (e.g., Tamis-LeMonda et al., 2024).

We created the Dual Language Learners English-Mandarin (DLL-EM) Inventories—with fully matched translation equivalents—to assess the vocabularies of infants/toddlers exposed to both English and Mandarin. An increasing number of children throughout the world are exposed to English and Mandarin from an early age, and Chinese Americans comprise the largest Asian origin group in the United States (Pew Research Center, 2025). Over three million Chinese Americans report speaking dialects of Chinese at home (e.g., Mandarin, Cantonese, etc.), with Mandarin being the most commonly spoken (U.S. Census Bureau, 2019). We investigated the psychometric properties of the newly created inventories (Study 1) and interviewed a test-case sample of Chinese American U.S. mothers on the vocabularies of their English-Mandarin dual language learning toddlers (Study 2).

### 1.2. The Need for Vocabulary Inventories with Full Translation Equivalents

Exposure to multiple languages is the norm rather than the exception worldwide (Grosjean, 2010; Harris & Nelson, 1992; United Nations Educational, Scientific and Cultural Organization, 2024), with dual language learners comprising a growing percentage of U.S. children. However, CDI language adaptations were not developed to assess the vocabularies of dual language learners, and bilingual children’s competencies should not be assessed through a monolingual lens (Genesee, 2022; Hoff, 2021).

Quantifying vocabulary size with a single CDI inventory does not credit bilinguals for the words they know in the other language, risking the impression that bilinguals have smaller vocabularies than monolinguals (e.g., Giguere & Hoff, 2022; Hoff et al., 2012; Thordardottir et al., 2006). One solution is to use two original CDI forms; however, such an approach poses problems as well, because words on the different language forms have minimal overlap (i.e., translation equivalents; Pearson et al., 1993, 1997). Although a lack of concept overlap is not an issue for quantifying the vocabularies of monolingual children (e.g., an English-speaking child in the United States or a Mandarin-speaking child in China), it raises concerns when applied to infants exposed to multiple languages. Non-overlapping items prevent researchers from accurately quantifying bilingual children’s *conceptual vocabulary*—the number of unique concepts a child knows across two languages (e.g., ‘cat’ and ‘猫’ are a single concept, but ‘猫’ and ‘dog’ are unique concepts)—because researchers must rely on a small subset of words to estimate conceptual vocabulary. Consequently, theoretical questions around mutual exclusivity (i.e., whether dual-language-learning infants are likely to have the word for a specific concept in one or both languages) cannot be addressed.

Similarly, estimates of bilingual children’s *total vocabulary* (i.e., summing words across the two language forms) may be problematic when using inventories with little conceptual overlap. Calculations of total vocabulary may overestimate the vocabularies of dual language learners relative to the vocabularies of monolingual infants because the two combined forms include more concepts than either CDI form. For example, if one CDI form asks if a child says ‘dog’ and the other asks if the child says ‘cat’ (in a different language), the application of the two forms will ask about cat *and* dog, whereas a single form would only ask about one. Indeed, the vocabulary sizes of bilingual children are sometimes larger than those of monolingual children because total vocabulary measures include more lexical forms across two languages relative to one (Pearson et al., 1993; Junker & Stockman, 2002; Core et al., 2013). Essentially, the size and direction of monolingual–bilingual differences may reflect the measurement approach itself rather than inherent linguistic differences (Byers-Heinlein et al., 2024).

Our aims were to (1) develop and (2) validate a new measure for assessing the vocabulary of English-Mandarin dual language learners, and to (3) administer the new inventory to a case sample to quantify infants’ vocabularies in both languages. In doing so, we addressed the urgent need for valid vocabulary inventories with fully matched concepts for English-Mandarin dual-language learners. In Study 1, we developed the DLL-EM Inventories based on existing English and Mandarin CDI short forms normed on data from monolingual infants/toddlers and tested their psychometric properties and convergent validity. In Study 2, we evaluated the feasibility of administering the DLL-EM Inventories and quantified the resulting vocabularies (in English, Mandarin, and both languages) for a case sample of dual-language-learning toddlers. We compared toddlers’ English and Mandarin vocabularies from the DLL-EM Inventories to estimated data that would be generated from two original CDI forms.

## 2. Study 1: Inventory Development & Validation

### 2.1. Overview

Several CDI adaptations have been created in Mandarin (e.g., Hao et al., 2008; Liu & Tsao, 2010; Tardif et al., 2009), the most widely used being developed and normed on vocabulary data of infants (8–16 months) and toddlers (16–30 months) from Beijing, China. However, like other CDIs adapted for monolingual infants, vocabulary items on the Mandarin adaptations have minimal overlap with the American English CDI. Thus, we created the DLL-EM Inventories with full translation equivalents for concepts based on the American English (Fenson et al., 2007) and Beijing Mandarin (Hao et al., 2008; Tardif & Fletcher, 2008) forms. We evaluated the psychometric properties of the DLL-EM Inventories and examined convergent validity by comparing estimated scores from DLL-EM Inventories against scores from CDI short forms using data from Wordbank (Frank et al., 2016) and Web-CDI (DeMayo et al., 2022).

### 2.2. Method

#### 2.2.1. Creation of the DLL-EM Inventories

**Figure 1:**
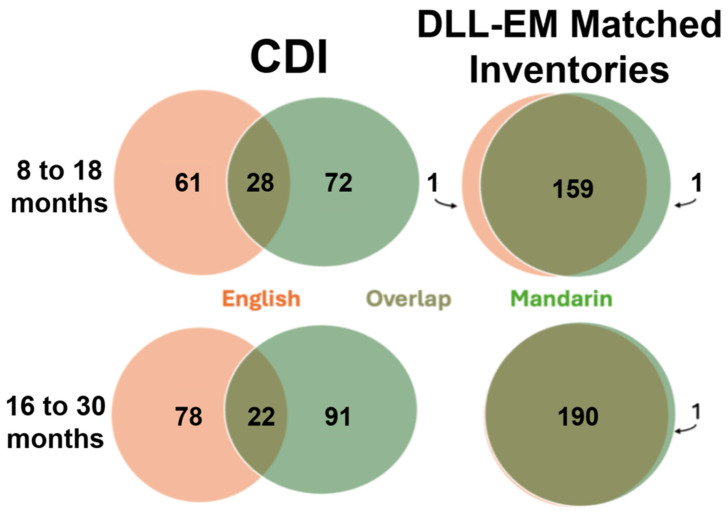
**Translation equivalents in two languages.** (**Top row**) Unique and overlapping concepts across original American English and Beijing Mandarin CDI short forms for Level 1 (top left) and Level 2 (bottom left). (**Bottom row**) Unique and overlapping concepts across English and Mandarin for the DLL-EM Level 1 (top right) and Level 2 (bottom right) Inventories.

Eight fluent English-Mandarin speaking researchers, representing different Mandarin dialectal regions (Beijing, Shanghai, Guangdong, Shandong, Taiwan, Hebei, Tianjin, Jilin, Hangzhou), compared Level 1 Mandarin and English CDI Words and Gestures short forms (106 and 89 words, respectively) and Level 2 Mandarin and English CDI Words and Sentences short forms (112 and 100 words, respectively). The comparison revealed minimal overlap between concepts on Mandarin and English forms for Level 1 and Level 2, respectively (Figure 1).

Researchers then identified translation equivalents for non-overlapping items to create two DLL-EM Inventories. The DLL-EM Level 1 is based on the CDI Words and Gestures short forms (8–16 months), and the DLL-EM Level 2 is based on the CDI Words and Sentences short forms (16–30 months). The DLL-EM Level 1 Inventory includes words from the original Mandarin and English CDI Words and Gestures short forms, supplemented with Mandarin translation equivalents for words that appear exclusively on the English Words and Gestures short form and English translation equivalents for words that appear exclusively on the Mandarin Words and Gestures short form. Similarly, the DLL-EM Level 2 Inventory includes words from the original Mandarin and English CDI Words and Sentences short forms, supplemented with Mandarin translation equivalents for words that appear exclusively on the English Words and Sentences short form and English translation equivalents for words that appear exclusively on the Mandarin Words and Sentences short form.

The English and Mandarin CDI long forms served as a starting point for identifying translation equivalents. For example, “eye” appears on the English CDI Words and Gestures short form but not on the Mandarin short form; however, its Mandarin equivalent “眼睛” appears on the Mandarin CDI Words and Sentences long form. Thus, “眼睛” was added to the DLL-EM Level 1 Inventory. When a translation equivalent did not appear in the long form of the other language, English-Mandarin bilingual researchers provided a translation equivalent through discussion (e.g., “翻” was translated as “*flip*”). Specifically, we included word variants where relevant to ensure that caregivers from different dialectal heritages, with unique cultural practices and linguistic features, would recognize the words and to avoid potential underreporting of children’s comprehension and production.

This process resulted in fully matched DLL-EM Inventories, with the exception of a few words. The final DLL-EM Level 1 Inventory contained 159 translation equivalents, with two unmatched items without direct translation equivalents—“你拍一,我拍一” and “patty cake”. These items reflect culturally specific children’s games adapted from the original CDI forms, rather than translation equivalents. Importantly, several concepts involved asymmetrical lexical mappings between English and Mandarin (i.e., items were collapsed into two-to-one mappings to align concepts across languages instead of one-to-one mappings). For example, the English pronouns *I* and *me* both correspond to the single Mandarin pronoun “我”. Similarly, multiple Mandarin kinship terms map to a single English equivalent: “哥哥” and “弟弟” correspond to *brother*, “姐姐” and “妹妹” correspond to *sister*, and “阿姨” and “姑姑” correspond to *aunt*. The Level 2 Matched Inventory contained 190 translation equivalents with 1 unmatched item (“中国”). Importantly, the resulting inventories included the same semantic categories (e.g., food, household items, body parts) and word classes (e.g., nouns, verbs, adjectives, prepositions, pronouns, and articles) represented in the original English and Mandarin short forms. Moreover, the DLL-EM Inventories retained all words on the original short forms—to allow researchers to generate percentile scores (if desired for each language) and additional translation equivalents in each language.

#### 2.2.2. Data Used for Evaluating Psychometric Properties & Testing Validity

To examine the psychometric properties and test the validity of the DLL-EM Level 1 Inventories, we leveraged CDI data on the vocabularies of monolingual English-speaking infants aged 12 to 18 months (pulled from Wordbank) and monolingual Mandarin-speaking infants aged 12 to 16 months (Hao et al., 2008;^1^ data pulled from Web-CDI). The English dataset consisted of 1650 caregiver reports on the English CDI Words and Gestures Form. The Mandarin dataset consisted of 230 caregiver reports from the Mandarin CDI Words and Gestures Form, which yielded a combined Level 1 sample of 1880 caregiver reports (Table 1).

To examine the psychometric properties and test the validity of the DLL-EM Level 2 Form, we used CDI data from monolingual English-speaking children aged 16 to 30 months (pulled from Wordbank) and monolingual Mandarin-speaking children aged 16 to 30 months. The English dataset consisted of vocabulary reports for 6480 toddlers from the English CDI Words and Sentences Form (pulled from Wordbank). The Mandarin dataset consisted of vocabulary reports for 1056 toddlers from the Mandarin CDI Words and Sentences Form (Tardif et al., 2009; data pulled from Wordbank). The combined Level 2 sample for English and Mandarin consisted of vocabulary reports for 7536 toddlers. Due to substantial missing data on sex and race in the Wordbank dataset, we report the total sample size for each group, without breakdowns by demographic variables.

#### 2.2.3. Examining Item-Level Psychometric Properties

**Figure 2:**
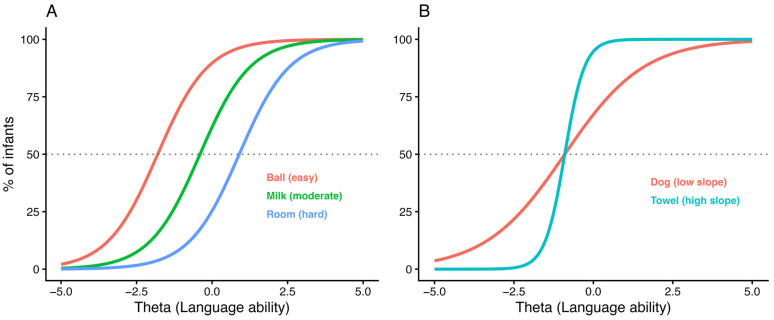
**Item Characteristic Curves.** Item Characteristic Curves (ICCs) illustrating item difficulty and discrimination based on Item Response Theory (IRT): In the IRT framework, the latent trait of language ability (theta) is standardized to have a mean of 0 and a standard deviation of 1. (**A**) **Difficulty parameter**: This panel displays ICCs for three example words that differ in difficulty. Note that the ICCs are illustrative rather than exact. The relative ordering of the words reflects the observed difficulty estimates. Words such as Ball (easy) appear to the left, indicating they are known by infants with lower language ability, whereas words like bedroom (hard) appear to the right, indicating they are acquired by infants with higher language ability. (**B**) **Discrimination parameter**: This panel displays ICCs for two items that differ in discrimination. Towel (high slope) has a steep curve, meaning it sharply distinguishes between infants with different levels of language ability. In contrast, Dog (low slope) has a more gradual curve, indicating it provides less discrimination between ability levels.

We examined the psychometric properties of the DLL-EM using Item Response Theory (IRT). Following DLL-ES (Tamis-LeMonda et al., 2024), we used the IRT intercept parameter (*d*) as an index of item easiness, with higher *d* values indicating easier items. In the example of vocabulary, IRT allows researchers to model the probability of an infant/toddler understanding or producing a given word across varying levels of language ability, which can be depicted with Item Characteristic Curves (ICCs). Such analyses yield information on the difficulty and discrimination of each word—namely, the point along the ability continuum at which a word is typically acquired. More difficult words appear further to the right on the ICC. For example, as illustrated in Figure 2 (Panel A), words that are acquired earlier, such as “ball”, appear toward the left of the curve, whereas more difficult words, such as “bedroom”, appear to the right. IRT analyses also provide information on item discrimination, or how effectively a word differentiates between children with varying vocabulary skills. Words with a steeper slope provide better discrimination of participants’ vocabulary abilities compared to words with a flatter slope. As demonstrated in Figure 2 (Panel B), the word “towel” has a steeper slope than “dog”, indicating that “towel” is better able to distinguish among children at different levels of vocabulary development.

Specific to the current study, we tested whether the English and Mandarin DLL-EM Inventories were comparable in difficulty using 2-parameter logistic Item Response Theory (IRT) models. That is, translation equivalents do not guarantee that the word for a specific concept has the same difficulty in both English and Mandarin because other factors such as phonological complexity and word frequency in each language also determine word difficulty (Brysbaert & Ghyselinck, 2006; Sosa & Stoel-Gammon, 2012). Differences in the difficulty of translation equivalents were calculated as the squared difference of the two difficulty estimates (i.e., mean difficulty of English form − mean difficulty of Mandarin), with higher values indicating greater differences in difficulty (e.g., Kachergis et al., 2022; Tamis-LeMonda et al., 2024). For example, the Mandarin word “牛奶” has a lower difficulty (dMN = −3.67) than its English counterpart “milk” (dEN = −1.36), so the squared difference between the two words is large (−3.67 − −1.36)^2 = 5.34. Through this item-level analysis, we identified word pairs with noticeable discrepancies in difficulty. To further contextualize differences at the aggregate level, we also compared the overall mean difficulties of English and Mandarin items within both the DLL-EM forms and the original CDI forms.

#### 2.2.4. Testing Convergent Validity

We conducted six IRT models to compare DLL-EM Inventories with the CDI English long-form scores and the CDI Mandarin long-form scores available on Wordbank and Web-CDI. Specifically, two models (English and Mandarin) examined the validity of comprehension scores by comparing the DLL-EM Level 1 Inventory with CDI long-form scores. Another two models assessed the validity of production scores within the same age group by evaluating the association between DLL-EM Level 1 Inventory and CDI long-form scores. Two additional models investigated production scores for older children by comparing the DLL-EM Level 2 Inventory with CDI long-form scores.

To further assess how well the DLL-EM Inventories predict CDI long-form scores compared to the predictive accuracy of original CDI short forms for CDI long-form scores, we conducted an additional six IRT models on association between the original CDI short forms and CDI long-form scores. This step aided interpretation of comparisons between the DLL-EM Inventories and CDI long forms. Specifically, two models (English and Mandarin) compared infants’ Level 1 comprehension based on original CDI Level 1 short-form comprehension scores to estimates from CDI long-form scores. Another two models (English and Mandarin) compared infants’ Level 1 production on original CDI Level 1 short forms to production on CDI long-form scores. Additionally, two models (English and Mandarin) compared toddlers’ Level 2 production based on original CDI Level 2 short-form scores to production on CDI long-form scores (Level 2 inventories assess production only). Based on the twelve models for DLL-EM Inventories, we tested whether (1) DLL-EM scores correspond to, underestimate, or overestimate CDI long-form scores, and whether these patterns are comparable to those observed for the original CDI short forms, and (2) DLL-EM scores are associated with CDI long-form scores to inform on convergent validity.

### 2.3. Results

#### 2.3.1. Item Level Difficulty

Analyses of item difficulty revealed discrepancies in aggregate word difficulty between English and Mandarin inventories (notably for Level 1). However, these differences were also present in the original English and Mandarin CDI forms from which the DLL-EM words were drawn (see Table 2). Specifically, in the original Level 1 CDI long forms, Mandarin words were on average more difficult than English words (dEN − dMN = −4.81 logits; higher values = harder), and similar patterns were observed for the original Level 1 CDI short forms (dEN − dMN = −4.20). These differences naturally propagated to the DLL-EM Level 1 form (dEN − dMN = −5.05). Filtering out the 8 most mismatched pairs modestly reduced this discrepancy (dEN − dMN = −4.57), but a cross-linguistic difference remained.

We identified eight pairs in the DLL-EM Level 1 form that exceeded a 1.5 SD cutoff for the squared difficulty difference. The Mandarin words may have been harder than their English translation equivalents due to their phonological complexity: Most words were bisyllabic with complex tonal demands (e.g., 洗澡/bath, 老鼠/mouse, both requiring Tone 3 sandhi), whereas all English translation equivalents were monosyllabic. Thus, removing a limited number of highly discrepant word pairs did not substantially improve cross-language alignment. As such, retaining pairs of words that differ in difficulty is consistent with prior work on matched bilingual inventories (Tamis-LeMonda et al., 2024).

In contrast, Level 2 forms yielded closer comparability across languages than did Level 1 forms (although English words were slightly more difficult than Mandarin words at this level). The Level 2 DLL-EM form difference was dEN − dMN = 2.86. This difference was similar to that of the original Level 2 CDI long forms (dEN − dMN = 3.21) and short forms (dEN − dMN = 2.45). The filtered (12 pairs excluded) DLL-EM Level 2 form did not improve cross-linguistic balance (dEN − dMN = 3.00).

#### 2.3.2. Convergent Validity

**Figure 3:**
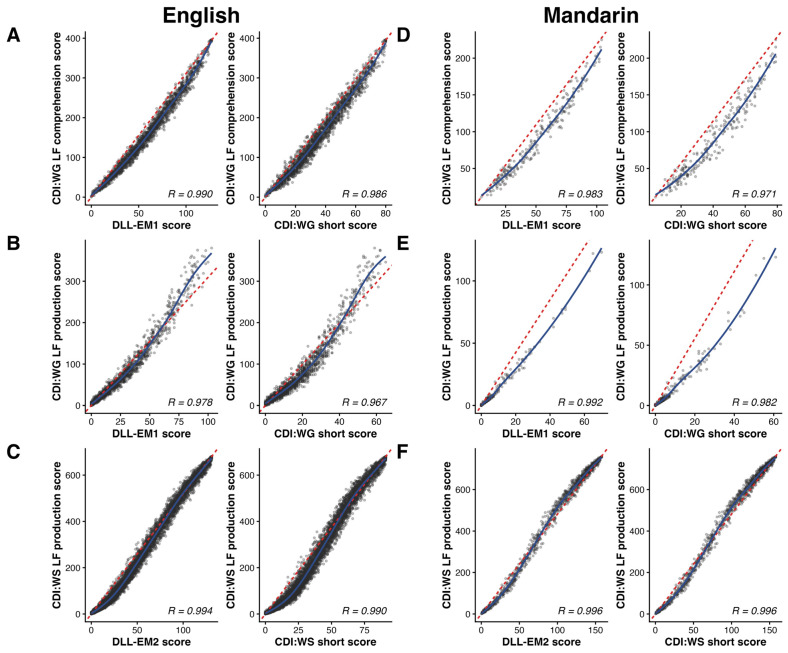
**Item-Response Theory (IRT) Results**. Estimates of Dual Language Learners English–Mandarin (DLL-EM) Inventories and Communicative Development Inventory (CDI) short-form scores from CDI long-form data in Wordbank and Web-CDI. Gray dots represent individual children. Blue solid lines represent the estimated relation between DLL-EM or CDI short-form scores and CDI long-form scores. Red dotted lines represent the expected proportional score based on the number of items in each short form relative to the CDI long form. Blue lines above the red dotted lines indicate overestimation of CDI long-form scores, whereas blue lines below the red dotted lines indicate underestimation. Pearson correlations are shown in each panel to summarize the association between estimated short-form scores and CDI long-form scores. (**A**) Estimated English comprehension scores for DLL-EM1 Inventories and CDI Words & Gestures (WG) short form. (**B**) Estimated English production scores for DLL-EM1 Inventories and CDI WG short form. (**C**) Estimated English production scores for DLL-EM2 and CDI Words & Sentences (WS) short form. (**D**) Estimated Mandarin comprehension scores for DLL-EM1 and CDI WG short form. (**E**) Estimated Mandarin production scores for DLL-EM1 and CDI WG short form. (**F**) Estimated Mandarin production scores for DLL-EM2 and CDI WS short form.

The DLL-EM Level 1 and Level 2 Inventories demonstrated strong convergent validity for both languages when compared to original English and Mandarin CDI long forms. The correlation coefficients ranged from *r* = 0.98 to 0.99 for the six models (Level 1 comprehension and Level 1 production, each for English and Mandarin; Level 2 production for English and Mandarin), indicating near-perfect associations (Table 3). The DLL-EM Level 1 Inventory for Mandarin vocabulary slightly underestimated comprehension and production scores when tested against the CDI Mandarin long forms (Figure 3). Still, the underestimation did not differ from predicted scores based on original CDI Mandarin Level 1 short-form scores.

### 2.4. Discussion Study 1

The DLL-EM Inventories predicted CDI long-form scores with near-perfect accuracy that was comparable to that of the original CDI short forms in monolingual English and Mandarin CDI datasets. The preliminary evidence for strong validity of the DLL-EM Inventories offers benefit over two original forms, given its fully matched items across English and Mandarin: Researchers can examine overlap in the words children understand and produce based on a larger set of words than permitted with original forms. This near-perfect association was maintained even though the English and Mandarin translation-equivalent items for the English and Mandarin differed in difficulty for the Level 1 forms. However, differences in difficulty were not unique to the DLL-EM Inventories; rather, they mirrored the substantial cross-linguistic differences already present in original English and Mandarin CDI short forms from which the items were drawn.

English and Mandarin differ widely in phonological complexity, morphological structure, frequency distributions, and early acquisition patterns, making perfect difficulty alignment neither expected nor always attainable. Thus, although comparisons of raw vocabulary scores across languages are useful, researchers must be cautious in interpreting an infant’s production of specific words that differ in critical ways in linguistic characteristics. Here, we retained all items to provide researchers with flexibility to exclude items that diverge in difficulty depending on their goals.

Note that DLL-EM scores in Study 1 are estimated from monolingual CDI data rather than from actual bilingual children. Similarly, the DLL-ES (Tamis-LeMonda et al., 2024), a parallel instrument in English-Spanish, also relied on monolingual data for its validation. This reflects a field-wide constraint: No publicly available bilingual English-Mandarin dataset exists, including the Wordbank. Therefore, how well estimates reflect true bilingual vocabulary can only be tested when additional bilingual data become available.

## 3. Study 2: A Case Sample of DLL-EM

### 3.1. Overview

In an exploratory study, we evaluated the feasibility of the DLL-EM Inventories for assessing bilingual toddlers’ vocabulary development in a sample of Mandarin-English dual-language learners. We quantified administration time and differences among toddlers in total and conceptual vocabulary size, distribution of words and concepts across English and Mandarin, and the degree to which concepts in one language overlapped with concepts in the other (i.e., mutual exclusivity). Relatedly, we compared data generated by the DLL-EM Inventories to estimated data that would be generated through administration of two original CDI short forms. Given the exploratory nature of the study and small sample, we were unsure about how vocabulary data from the DLL-EM Inventories with full translation equivalents would align with estimates from original CDIs.

### 3.2. Participants

Participants were 30 mothers of 18- to 30-month-old English–Mandarin DLL toddlers (10 female; *M* age = 23.9 months; *SD* age = 3.5 months), living in the NYC metropolitan area. Participants were drawn from a local database, which recruited families from hospitals, well-baby visits, and community organizations. Inclusionary criteria included mothers who reported speaking to their toddlers in Mandarin at home over 80% of the time, and toddlers being full-term at birth and typically developing without a formal diagnosis.

Mothers averaged 35.5 years (range = 30–43 years). Almost all mothers reported being born in China (*n* = 28 born in China; *n* = 2 born in the United States), and U.S. residency ranged from 4 to 26 years (*M* = 12; *SD* = 5). All toddlers were born in the United States. Ten mothers reported having graduate or professional degrees, nine reported some college or bachelor’s degree, two reported a high school degree or equivalent, and eight reported fewer years than high school. Nineteen mothers reported working for pay, and nine mothers reported not working for pay. Mothers signed written consent forms for their participation and received $100 digital gift cards.

### 3.3. Procedures

A fluent English-Mandarin bilingual researcher interviewed mothers in the language mothers were most comfortable speaking. The researcher gave examples of what was meant by “producing a word” and explained that the child’s pronunciation of the word must be used consistently but did not have to be standard pronunciation (e.g., “baba” for bottle was credited; see Databrary link redacted for review for full protocol). The researcher read each item on the DLL-EM Level 2 Inventories, beginning with words from the original CDI short forms, followed by the added Mandarin and English translation equivalents, always starting with words in the child’s dominant language. Mothers had a copy of the word lists in hand to facilitate following along.

### 3.4. Results

#### 3.4.1. DLL-EM Inventories Show Feasibility and Low Burden Administration Time

Administration of the DLL-EM Level 2 Inventories indicated low time burden. The DLL-EM Inventories averaged less than 15 min to administer, including instructions (*M* = 14 min 49 s; *range* = 7–31 min).

#### 3.4.2. DLL-EM Inventories Yield Large Differences Among Toddlers in Mandarin and English Vocabularies

Even in this test case, differences among toddlers’ bilingual vocabularies were striking. Mothers reported that toddlers produced an average of 76.79 total words (*SD* = 72.05), with vocabularies ranging from 1 to 227 words. Total vocabulary counts credited toddlers for each form they produced in a duplet—that is, a pair of translation-equivalent forms for the same concept, such as dog and 狗 were credited as two words. Of the 381 total words on the DLL-EM Level 2 Inventories, at least 356 words were produced by at least one toddler. Specifically, 173 of 190 English words and 183 of 191 Mandarin words were produced by at least one toddler.

Toddlers also differed in their number of total concepts. Toddlers produced an average of *M* = 62.32 total concepts (*SD* = 51.63), with a range from 1 to 166 (i.e., crediting production of any word in a translation-equivalent pair as one concept). Of the 191 concepts on the DLL-EM Inventories, at least 188 were produced by at least one toddler. There were three concepts no child produced—“pitiful/可怜”, “(game) piece/棋子” and “would/肯”. “would” from “would/肯” is pulled from the English CDI short form, and “可怜” and “棋子”, from “pitiful/可怜” and “(game) piece/棋子”, are words from the Beijing Mandarin CDI short form.

#### 3.4.3. DLL-EM Inventories Yield Large Differences Among Toddlers in Word Distribution Across Languages

**Figure 4:**
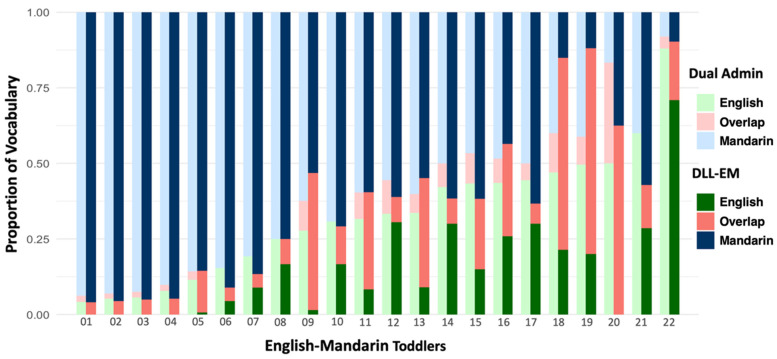
**Vocabulary concepts in English and Mandarin based on DLL-EM Inventories versus estimates from original English and Mandarin CDI forms**. Each pair of bars represents a toddler. Bars present the proportions of unique English concepts (green bars), unique Mandarin concepts (blue bars), and overlapping concepts (red bars). The light-shaded bars (left side) for each toddler are estimates based on the administration of original English and Mandarin short forms. Dark shaded bars (right side) are based on use of matched DLL-EM Level 2 Inventories. Toddlers < 5 total concepts on either the dual administration or the DLL-EM are not included in the figure.

Toddlers also differed in their distribution of words across English and Mandarin (Figure 4). Toddlers ranged from 0% to 76% of their words being in English (with total vocabulary as the denominator; *M* = 23%; *SD* = 19). Similarly, toddlers ranged from 24% to 100% of their words being in Mandarin (with total vocabulary as the denominator; *M* = 77%; *SD* = 19).

#### 3.4.4. Data Generated by DLL-EM Inventories Differ from Those of Two Original CDIs

Notably, the DLL-EM Inventories’ use of full translation equivalents yielded a very different portrayal of toddlers’ vocabularies in English and Mandarin compared to data that would result from dual administration of the original English and Mandarin short forms. Specifically, examination of the precise words toddlers produced in each language revealed that mutual exclusivity was not the norm when using the DLL-EM (versus original CDI short forms), at least in this small case sample of families. Rather, toddlers were likely to have words in both languages. Specifically, of the 190 English and Mandarin matched concepts (with one word left unmatched), 125 concepts (65%) were produced in both English and Mandarin by at least one toddler. At an individual level, toddlers varied in the percent of words representing overlapping concepts, ranging from 0% to 68% (*M* = 17.6%; *SD* = 20.4%). In contrast, overlapping concepts would be minimal if the two original CDI short forms were used: A very low percent of overlapping concepts were seen for original forms (i.e., 22 words compared to the 190 seen here).

Figure 4 depicts unique English, unique Mandarin, and overlapping words for individual toddlers based on a dual administration of the DLL-EM Inventories compared to what would be generated by the two original CDI forms (i.e., words on the non-matched American English and Beijing Mandarin short forms). Side-by-side comparisons yield unique portrayals of the vocabularies of individual children. For example, a dual-administration assessment of toddler #10’s vocabulary would present little concept overlap, a pattern that might result in conclusions of nearly full mutual exclusivity. However, the same child’s scores on the matched DLL-EM Inventories indicated substantial concept overlap (60% of English words in the child’s vocabulary had a matched concept in Mandarin). Inventories with non-overlapping concepts risk inferring that toddlers have words in Mandarin only or English only (see Figure 4). But when using matched forms, the overlap becomes apparent.

#### 3.4.5. Distribution and Totals

The relatively high proportion of overlapping words in two languages resulted in differences in the distribution of children’s words across Mandarin and English languages compared to original short forms. Specifically, the matched DLL-EM Inventories revealed a lower proportion of English words (*M* = 23%, *SD* = 18%) compared to the proportion obtained from a dual administration of the original CDIs (*M* = 30%, *SD* = 20%), though this difference was not statistically significant, *t*(29) = −1.03, *p* = 0.31. Conversely, the DLL-EM Inventories yielded a greater proportion of Mandarin words (*M* = 77%, *SD* = 18%) compared to the estimated proportion obtained with dual administration of the original CDIs (*M* = 60%, *SD* = 30%), *t*(29) = 2.45, *p* < 0.05. By testing words with matched translation equivalents, the DLL-EM Inventories credited Mandarin words that might otherwise be captured only in English on the original forms (and vice versa)—thus providing a fuller picture of each child’s bilingualism.

### 3.5. Discussion Study 2

Study 2 provided illustrative data on administration of the DLL-EM relative to two original short forms. Administration of the DLL-EM revealed low time burden. In contrast, administering separate Mandarin and English CDI original forms would require caregivers to report on an unwieldy number of items. However, the DLL-EM was quick and feasible to administer, averaging under 15 min for the full matched inventory.

Beyond feasibility, the DLL-EM Inventories yielded substantial differences among toddlers in total vocabulary, conceptual vocabulary, and the distribution of words between Mandarin and English compared to the dual administration of CDI short forms. Most centrally, findings from this exploratory sample indicate that matched vs. unmatched forms may result in different portrayals of dual language learners’ vocabulary. As one example, the DLL-EM revealed substantial concept overlap between words in each language in this sample, supporting prior findings that mutual exclusivity is not a rigid constraint, but a flexible, experience-dependent process (Davidson et al., 1997; Byers-Heinlein & Werker, 2009).

Of course, the study is limited in its small sample of participants, from a single geographic region, with specific demographic characteristics. Thus, the high proportion of Mandarin words likely reflects the Mandarin-dominant home language environments and predominantly first-generation immigrant status of the sample of mothers. Other patterns may likely arise when using the forms in other contexts (e.g., English-dominant homes or second-generation immigrant families).

## 4. General Discussion

We created the DLL-EM Inventories—a parent-report instrument for assessing the early vocabularies of Mandarin-English dual language learners and provided strong initial evidence for their psychometric properties and convergent validity. The DLL-EM Inventories, which contain translation equivalents for all words, allow researchers to assess the extent to which words in a child’s vocabulary are shared between languages, thus offering a full picture of early bilinguals’ language development. Moreover, the DLL-EM Inventories allow researchers to generate percentile scores based on original English and Mandarin short forms by pulling from subsets of words in the Inventories (full DLL-EM Inventories are available as Supplementary Materials). Centrally, a major contribution of the DLL-EM Inventories is the ability to quantify conceptual overlap in bilingual children’s vocabularies. The DLL-EM Inventories include 159 and 190 matched concepts, for Levels 1 and 2, respectively, in contrast to the original CDI forms that share only 28 and 22 items in the two levels.

Notably, the development and validation of the DLL-EM—and evidence of contrasting findings around vocabulary development based on matched vs. nonmatched forms—have scientific relevance for many theoretical questions. Findings speak to topics of mutual exclusivity (are infants/toddlers biased to use a single word for a concept?; Weatherhead et al., 2021; Houston-Price et al., 2010; Bleijlevens et al., 2025), conceptual vocabulary (how large are toddlers’ conceptual vocabularies when considering words in both languages?; Byers-Heinlein et al., 2024; Siow et al., 2023), developmental trajectories in vocabulary growth (how much “overlap” is seen in infants and toddlers at different ages and as vocabularies grow?; Tan et al., 2024; Gámez et al., 2023), how demographic and contextual factors influence vocabulary growth in dual-language-learning children (Luo et al., 2021; Goodrich et al., 2021), and the extent to which acquiring the word for a concept in L1 facilitates the acquisition of a word for the same concept in L2 (Floccia et al., 2020; Tan et al., 2024; Siow et al., 2025).

### 4.1. The DLL-EM Inventories Show Strong Psychometric Properties and Convergent Validity

The DLL-EM Inventories showed strong psychometric properties: Item Response Theory (IRT) analyses revealed that the DLL-EM Inventories are psychometrically robust and show near-perfect correlations with the original CDI long forms, indicating strong convergent validity. Specifically, the DLL-EM Inventories’ word difficulty and discrimination parameters were well aligned with the original CDIs across English and Mandarin. Of course, a limitation is that our comparisons and validation of the DLL-EM were (by necessity) against vocabulary data drawn from original CDIs developed on monolingual children. As use of the DLL-EM grows, so will available data on the vocabularies of Mandarin-English dual language learners.

### 4.2. The DLL-EM Shows Low Burden and Novel Insights into Early Bilingual Vocabulary

A test-case sample revealed that the DLL-EM Inventories are easily administered with little time burden to parents, relative to the time it would take to administer both an English and a Mandarin short-form CDI. Moreover, diverging patterns in toddlers’ conceptual overlap when comparing DLL-EM data to estimates generated from original forms show how choice of inventory can impact inferences about word learning, such as mutual exclusivity—which is shaped by bilingual children’s experience with lexical overlap and translation equivalents (e.g., Bosch & Ramon-Casas, 2014; Kalashnikova et al., 2015). Although the high conceptual overlap in toddlers’ words (i.e., a word for the concept in both languages) does not refute the role of mutual exclusivity in early language acquisition (particularly for monolingual infants). Rather, findings challenge interpretations about dual-language-learners that may arise from assessments in the absence of full translational equivalents.

In this sample, the DLL-EM Inventories captured a higher proportion of Mandarin vocabulary than administering separate CDIs. The inventories also yielded large differences among toddlers in the composition of their vocabularies across the two languages. Between-toddler differences likely reflect meaningful variation in language exposure, context of use, or community language practices and underscore the unique developmental trajectories of DLL children’s vocabulary growth. As others have argued, bilingualism is not two monolinguals in one brain—it is its own linguistic experience (e.g., De Houwer, 2009; Genesee, 2022; Hoff, 2021).

### 4.3. Contextualizing Inventories of Child Vocabulary

There is a clear need for vocabulary assessments created for dual language learners (Tamis-LeMonda et al., 2024; Weisleder et al., 2024). We were sensitive to the reality that creating matched inventories requires much more than identifying direct translations (Marchman & Dale, 2023). It involves aligning word difficulty across languages, accounting for cultural specificity, and navigating dialectal variation (Erkut, 2010; Peña, 2007). Thus, in developing the DLL-EM Inventories, decisions were made to adapt rather than “simply” translate^2^.

Furthermore, although we tested the DLL-EM in a case sample of immigrant Chinese families in New York City, the DLL-EM Inventories have the potential for broader application across national and international contexts where English and Mandarin are commonly used. In the United States, states like California and New York demonstrate strong demand for bilingual Mandarin-English tools, reflecting both population demographics and educational needs (Hammer et al., 2011, 2014; U.S. Census Bureau, 2019). Beyond the United States, the DLL-EM Inventories are well-suited for parts of mainland China, where early English exposure—via media, schooling, and extracurriculars—is increasingly common (Feng, 2012; Hu & McKay, 2012; Ministry of Education of the People’s Republic of China, 2001; Sun et al., 2016). The tool also holds promise in regions like Australia, Canada, Malaysia, Taiwan, Singapore, and the United Kingdom, where English-Mandarin bilingualism is prevalent and shaped by diverse sociolinguistic dynamics (Poston & Wong, 2016). Given its global relevance, the implementation of DLL-EM Inventories can function not only as a practical tool but also as a flexible framework—enabling researchers to tailor and adapt the matched inventories to different sociocultural contexts and interpret scores to reflect children’s everyday language experiences.

Oral administration is also recommended to facilitate caregivers’ understanding of words on the inventories and researchers’ dialect-specific adaptations. In New York City, several families encountered challenges with specific word types—particularly classifiers and translation equivalents that did not align with their daily language practices. In such cases, omissions or clarifications may be warranted to ensure cultural appropriateness.

Finally, when paired with detailed information about children’s experiences, such as household language use, caregiver language preferences, socioeconomic status, migration history, and community language exposure (Place & Hoff, 2011, 2016; Yoshikawa et al., 2016), vocabulary tools like the DLL-EM Inventories allow researchers and practitioners to better capture the wide heterogeneity that characterizes early vocabulary growth across two or more languages (Freeman & Schroeder, 2022; Paradis, 2023).

## 5. Conclusions

The DLL-EM Inventories offer a valid, low-burden, and culturally responsive approach to assessing early bilingual vocabulary in English-Mandarin bilingual children. Translation equivalents across languages streamline assessment, reduce caregiver burden, and, most centrally, expand the theoretical and methodological tools available to researchers and clinicians. In a multilingual world, assessing the language development of children through a monolingual lens is insufficient. In response, the DLL-EM Inventories enable researchers to document the words toddlers acquire for unique and shared concepts across languages during a period of awe-inspiring vocabulary growth.

## Figures and Tables

**Table 1:** **Overview of CDI Forms and DLL-EM Inventories Across Levels and** **Languages.**

Forms	Level	Languages	Number of Words	Source of Data
CDI Forms	Level 1 (Word & Gestures)	English	89	Wordbank
		Mandarin	106	Web-CDI
	Level 2 (Word & Sentences)	English	100	Wordbank
		Mandarin	112	Wordbank
DLL-EM Inventories	Level 1 (Word & Gestures)	English	161	Wordbank
		Mandarin	163	Web-CDI
	Level 2 (Word & Sentences)	English	190	Wordbank
		Mandarin	191	Wordbank

**Table 2:** **Mean Item Difficulty**. Mean Item Difficulty (d) for English and Mandarin Words Across Levels and Forms, Including DLL-EM Inventories, Original CDI Short Forms, and Long Forms from the Wordbank and Web-CDI Data.

Level	Form/Subset	Language	Mean d	Mean d Difference (English − Mandarin)
1	Long forms	English	2.23	−4.81
		Mandarin	7.04	
	Original CDI short forms	English	0.67	−4.20
		Mandarin	4.87	
	DLL-EM	English	0.41	−5.05
		Mandarin	5.46	
	Non-DLL1 words	English	2.53	−5.68
		Mandarin	8.21	
	Filtered DLL-EM	English	0.44	−4.57
		Mandarin	5.01	
2	Long forms	English	2.23	3.21
		Mandarin	−0.98	
	Original CDI short forms	English	1.83	2.45
		Mandarin	−0.62	
	DLL-EM	English	1.87	2.86
		Mandarin	−0.99	
	Non-DLL2 words	English	2.32	3.30
		Mandarin	−0.98	
	Filtered DLL-EM	English	1.69	3.00
		Mandarin	−1.31	

**Table 3:** **Convergent validity**. Correlations Between Scores on the CDI Long Forms and Estimated Scores on the Dual Language Learners English–Mandarin (DLL-EM) Inventories for English (Top Three Rows) and Mandarin (Bottom Three Rows).

Original CDI Long Forms	Language	*N*	*r* with CDI Short Form	*r* with DLL-EM Inventores
CDI: Words & gestures comprehension	English	1650	0.986	0.990
CDI: Words & gestures production	English	1650	0.967	0.978
CDI: Words & sentences production	English	6480	0.990	0.994
CDI: Words & gestures comprehension	Mandarin	230	0.971	0.983
CDI: Words & gestures production	Mandarin	230	0.982	0.992
CDI: Words & sentences production	Mandarin	1056	0.996	0.996

Note. Ns reflect the final analytic samples used in each analysis after applying language-specific age restrictions and excluding cases with missing data.

## Data Availability

Spreadsheets and scripts for data analysis are shared on Databrary (https://nyu.databrary.org/volume/1748).

## Notes

1 We used level 1 data from (Hao et al., 2008) due to the absence of Tardif level 1 data in Wordbank and Web-CDI.

2 For example, we paired kinship terms (e.g., “哥哥” and “弟弟” to “brother”), placed culturally equivalent but translationally non-equivalent items near each other in the list for comparison, but treated them as non-matching items (e.g., children’s games like “patty cake,” and “你拍一,我拍一”), adapted word choices to reflect cultural relevance in our context (e.g., changing “棋子 [chess piece]” to a more general “game piece”), and omitted translations for words less relevant in the U.S. context (e.g., excluding the English equivalent for “中国” when testing U.S.-based DLLs). These choices align with earlier adaptations—such as Tardif and Fletcher (2008)’s inclusion of “(game) piece/棋子” in the Beijing Mandarin forms, a concept U.S.-based parents reported their children did not produce, but a culturally relevant item in the Beijing context—and underscore the importance of creating matched inventories that are culturally and linguistically appropriate, rather than merely translationally equivalent.

## Supplementary Materials

The following supporting information can be downloaded at: https://www.mdpi.com/article/10.3390/bs16071143/s1, Supplementary Materials_DLL-EM Inventories. Supplementary materials can also be found at: (https://nyu.databrary.org/volume/1748).
