# Supplementary material for: Development of a Vocabulary Inventory for English-Mandarin Dual-Language-Learning Infants and Toddlers
Source: Behav Sci (Basel). 2026 Jul 8;16(7):1143. doi: 10.3390/bs16071143 (PMC13404754; doi:10.3390/bs16071143)
Supplement: Supplementary file 1 [file behavsci-16-01143-s001.zip › behavsci-4264781-supplementary.pdf]

Supplementary Materials: DLL-EM Forms

Table S1. DLL-EM Level 1 (Infant) Vocabulary List

Category	English Word	Source English	Mandarin Word	Source Mandarin
action_words	hit	LF English	打	SF Mandrain
action_words	touch	LF English	摸	SF Mandrain
action_words	walk	LF English	走	SF Mandrain
action_words	wait	SF English	等	LF Mandrain
action_words	break	SF English	弄坏	-
action_words	feed	SF English	喂	SF Mandrain
action_words	finish	SF English	做完	LF Mandrain
action_words	help	SF English	帮助	LF Mandrain
action_words	jump	SF English	跳	LF Mandrain
action_words	kick	SF English	踢	SF Mandrain
action_words	kiss	SF English	亲（一个）	SF Mandrain
action_words	push	SF English	推	LF Mandrain
action_words	sing	SF English	唱	LF Mandrain
action_words	smile	SF English	笑	LF Mandrain
action_words	eat	LF English	吃	SF Mandrain
action_words	fall	LF English	掉	SF Mandrain
action_words	look	LF English	看/瞅	SF Mandrain
action_words	put on (clothing)	LF English	戴	SF Mandrain
action_words	take	LF English	拿（过来）	SF Mandrain
action_words	want	LF English	要	SF Mandrain
action_words	hug	LF English	抱，搂	SF Mandrain
action_words	eat	LF English	吃饭	SF Mandrain
action_words	come	-	来	SF Mandrain
action_words	fly	LF English	飞	SF Mandrain
action_words	give	LF English	给	SF Mandrain
adjective	thirsty	LF English	渴	SF Mandrain
animals	bunny	LF English	小白兔/兔子	SF Mandrain
animals	horse	LF English	马	SF Mandrain
animals	dog	SF English	狗	SF Mandrain
animals	car	SF English	车	SF Mandrain
animals	bird	SF English	鸟	SF Mandrain
animals	duck	SF English	鸭子	SF Mandrain
animals	kitty	SF English	小猫	-
animals	lion	SF English	狮子	LF Mandrain
animals	mouse	SF English	老鼠	LF Mandrain
animals	cat	LF English	猫	SF Mandrain

**animals**	**chick**	-	小鸡	SF Mandrain
**body_parts**	**foot**	LF English	脚	SF Mandrain
**body_parts**	**eye**	SF English	眼睛	LF Mandrain
**body_parts**	**head**	SF English	头	LF Mandrain
**body_parts**	**leg**	SF English	腿	LF Mandrain
**body_parts**	**nose**	SF English	鼻子	SF Mandrain
**body_parts**	**tooth**	SF English	牙	LF Mandrain
**body_parts**	**hand**	LF English	手	SF Mandrain
**body_parts**	**mouth**	LF English	嘴（口）	SF Mandrain
**body_parts**	**back**	-	背	SF Mandrain
**body_parts**	**buttocks/ bottom (or word used in your family)**	LF English	屁股	SF Mandrain
**clothing**	**pants**	SF English	裤子	LF Mandrain
**clothing**	**shoe**	SF English	鞋	SF Mandrain
**clothing**	**hat**	SF English	帽子	SF Mandrain
**clothing**	**sock**	SF English	袜子	SF Mandrain
**clothing**	**clothes**	-	衣服	SF Mandrain
**descriptive_words**	**big**	SF English	大	LF Mandrain
**descriptive_words**	**broken**	SF English	坏了/破了	LF Mandrain
**descriptive_words**	**pretty**	SF English	漂亮/美	SF Mandrain
**descriptive_words**	**all gone**	SF English	没了	LF Mandrain
**descriptive_words**	**dark**	SF English	黑/暗	-
**descriptive_words**	**fast**	SF English	快	LF Mandrain
**descriptive_words**	**hurt**	SF English	疼/痛	LF Mandrain
**descriptive_words**	**soft**	SF English	软	LF Mandrain
**descriptive_words**	**dirty**	LF English	脏	SF Mandrain
**descriptive_words**	**hot**	LF English	烫	SF Mandrain
**descriptive_words**	**Great Job! Well done!**	-	真棒!	SF Mandrain
**descriptive_words**	**smelly**	LF English	臭	SF Mandrain
**descriptive_words**	**full**	LF English	饱	SF Mandrain
**descriptive_words**	**angry**	LF English	生气	SF Mandrain
**descriptive_words**	**good**	LF English	好	SF Mandrain
**food_drink**	**bread**	SF English	面包	LF Mandrain
**food_drink**	**water**	SF English	水	SF Mandrain
**food_drink**	**candy**	SF English	糖果	LF Mandrain
**food_drink**	**cereal**	SF English	麦片	LF Mandrain
**food_drink**	**cookie**	SF English	饼干	SF Mandrain
**food_drink**	**juice**	SF English	果汁	LF Mandrain

**food_drink**	**toast**	SF English	吐司	LF Mandrain
**food_drink**	**egg**	LF English	蛋	SF Mandrain
**food_drink**	**meat**	LF English	肉	SF Mandrain
**food_drink**	**milk**	LF English	牛奶	SF Mandrain
**food_drink**	**fish**	LF English	鱼	SF Mandrain
**food_drink**	**banana**	LF English	香蕉	SF Mandrain
**food_drink**	**drink**	LF English	喝	SF Mandrain
**food_drink**	**rice**	-	饭	SF Mandrain
**furniture_rooms**	**door**	LF English	门	SF Mandrain
**furniture_rooms**	**kitchen**	SF English	厨房	LF Mandrain
**furniture_rooms**	**chair**	SF English	椅子	LF Mandrain
**furniture_rooms**	**couch**	SF English	沙发	LF Mandrain
**furniture_rooms**	**table**	SF English	桌子	LF Mandrain
**furniture_rooms**	**bed**	LF English	床	SF Mandrain
**games_routines**	**please**	SF English	请	LF Mandrain
**games_routines**	**bath**	SF English	洗澡	LF Mandrain
**games_routines**	**hi**	SF English	你好	LF Mandrain
**games_routines**	**patty cake**	SF English	-	-
**games_routines**	**-**	-	你拍一，我拍一	SF Mandrain
**games_routines**	**night night**	SF English	晚安	-
**games_routines**	**good boy/girl**	-	乖	SF Mandrain
**games_routines**	**morning**	LF English	早上	SF Mandrain
**helping_verbs**	**don't**	SF English	不要	LF Mandrain
**household**	**clock/watch**	LF English	钟/表	SF Mandrain
**household**	**paper**	LF English	纸	SF Mandrain
**household**	**pillow**	LF English	枕头	SF Mandrain
**household**	**TV (television)**	SF English	电视	SF Mandrain
**household**	**bottle**	SF English	瓶子	SF Mandrain
**household**	**cup**	SF English	杯子	LF Mandrain
**household**	**dish**	SF English	菜	LF Mandrain
**household**	**lamp**	SF English	灯	SF Mandrain
**household**	**radio**	SF English	收音机	LF Mandrain
**household**	**spoon**	SF English	勺	SF Mandrain
**household**	**blanket**	SF English	被子	SF Mandrain
**household**	**bowl**	LF English	碗	SF Mandrain
**household**	**mirror**	LF English	镜子	SF Mandrain
**outside**	**tree**	LF English	树	SF Mandrain
**outside**	**flower**	SF English	花	SF Mandrain
**outside**	**plant**	SF English	植物	-

**outside**	**home**	SF English	家	SF Mandrain
**outside**	**moon**	SF English	月亮	LF Mandrain
**outside**	**rain**	SF English	(下) 雨	LF Mandrain
**outside**	**rock**	SF English	石头	LF Mandrain
**outside**	**sky**	LF English	天空	SF Mandrain
**people**	**brother**	LF English	弟弟	SF Mandrain
			哥哥	SF Mandrain
**people**	**daddy (or word used in your family)**	LF English	爸爸	SF Mandrain
**people**	**grandpa**	LF English	爷爷	SF Mandrain
**people**	**sister**	LF English	妹妹	SF Mandrain
			姐姐	SF Mandrain
**people**	**aunt**	LF English	阿姨	SF Mandrain
			姑姑	SF Mandrain
**people**	**Grandma (or word used in your family)**	SF English	奶奶	SF Mandrain
**people**	**mommy (or word used in your family)**	SF English	妈妈	SF Mandrain
**people**	**babysitter**	SF English	保姆	LF Mandrain
**people**	**girl**	SF English	女孩	LF Mandrain
**people**	**baby**	LF English	宝宝	SF Mandrain
**people**	**child's own name**	LF English	自己的名字	SF Mandrain
**people**	**child**	LF English	孩子/小孩/小朋友	SF Mandrain
**people**	**uncle**	LF English	叔叔	SF Mandrain
**prepositions_location**	**outside**	SF English	外面	SF Mandrain
**prepositions_location**	**away**	SF English	走开	LF Mandrain
**prepositions_location**	**under**	LF English	下面	SF Mandrain
**prepositions_locations**	**here**	-	这边	SF Mandrain
**prepositions_locations**	**on**	LF English	上（面）	SF Mandrain
**prepositions_locations**	**there**	LF English	那边	SF Mandrain
**prepostions_locations**	**out**	SF English	外面	LF Mandrain
**pronouns**	**I**	SF English	我	SF Mandrain
	**me**	SF English		
**pronouns**	**this**	LF English	这个	SF Mandrain
**quantifiers_articles**	**other**	SF English	其他	LF Mandrain

**quantifiers_article s**	**some**	SF English	(一)些	LF Mandrain
**quantifiers_article s**	**much**	LF English	多	SF Mandrain
**quantifiers_article s**	**one**	LF English	一	SF Mandrain
**quantifiers_article s**	**two**	LF English	二	SF Mandrain
**question_words**	**how**	SF English	怎么	LF Mandrain
**question_words**	**who**	SF English	谁	SF Mandrain
**question_words**	**where**	LF English	在哪里/在哪儿?	SF Mandrain
**sounds**	**vroom (car sound)**	LF English	嘀嘀（汽车声）	SF Mandrain
**sounds**	**meow**	SF English	喵（猫叫）	LF Mandrain
**sounds**	**ouch**	SF English	哎呦	SF Mandrain
**sounds**	**choo choo (train sound)**	SF English	呜呜 (火车声)	-
**sounds**	**uh oh**	SF English	呃哦	-
**sounds**	**woof woof**	LF English	旺旺（狗叫）	SF Mandrain
**time**	**today**	SF English	今天	LF Mandrain
**time_words**	**night**	SF English	晚上	SF Mandrain
**toys**	**ball**	SF English	球	SF Mandrain
**toys**	**book**	SF English	书	LF Mandrain
**toys**	**doll**	SF English	小娃娃	SF Mandrain
**vehicles**	**airplane**	LF English	飞机	SF Mandrain
**vehicles**	**stroller**	SF English	婴儿车 / 娃娃车（手推车）	- & LF Mandrain

Table S2. DLL-EM Level 2 (Toddler) Vocabulary List

Category	Word in English	Source English	Word in Mandarin	Source Mandarin
action_words	rub	-	揉	SF Mandarin
action_words	flip	-	翻	SF Mandarin
action_words	twist	-	拧	SF Mandarin
action_words	pat	-	拍	SF Mandarin
action_words	not allowed	-	不许	SF Mandarin
action_words	fall	LF English	掉	SF Mandarin
action_words	play	LF English	玩	SF Mandarin
action_words	sit	LF English	坐	SF Mandarin
action_words	have	LF English	有	SF Mandarin
action_words	give	LF English	给	SF Mandarin
action_words	send	LF English	送	SF Mandarin
action_words	wait	LF English	等	SF Mandarin
action_words	pick (in the context of pluck)	LF English	摘（揪）	SF Mandarin
action_words	remember	LF English	记（得）	SF Mandarin
action_words	use	LF English	用	SF Mandarin
action_words	swim	LF English	游泳	SF Mandarin
action_words	stick (to adhere)	LF English	贴	SF Mandarin
action_words	hide	LF English	躲	SF Mandarin
action_words	ask	LF English	问	SF Mandarin
action_words	tie	LF English	绑	SF Mandarin
action_words	stir	LF English	搅	SF Mandarin
action_words	try/try to	LF English	试	SF Mandarin
action_words	follow	LF English	跟	SF Mandarin
action_words	(to) swing	LF English	悠 / 荡	SF Mandarin & -
action_words	rip	SF English	撕	-
action_words	carry	SF English	背，端	LF Mandarin
action_words	chase	SF English	追	LF Mandarin
action_words	dump	SF English	倒（垃圾）	LF Mandarin
action_words	finish	SF English	做完	LF Mandarin
action_words	fit	SF English	适合	LF Mandarin
action_words	hug	SF English	抱，搂	LF Mandarin
action_words	like	SF English	喜欢	LF Mandarin
action_words	listen	SF English	听	LF Mandarin
action_words	shake	SF English	摇	LF Mandarin
action_words	taste	SF English	尝	LF Mandarin

**action_words**	**think**	SF English	想	LF Mandarin
**action_words**	**pretend**	SF English	假装	SF Mandarin
**action_words**	**wish**	SF English	希望	SF Mandarin
**animals**	**animal**	LF English	动物	SF Mandarin
**animals**	**peacock**	LF English	孔雀	SF Mandarin
**animals**	**mosquito**	LF English	蚊子	SF Mandarin
**animals**	**dinosaur**	LF English	恐龙	SF Mandarin
**animals**	**cat**	SF English	猫	LF Mandarin
**animals**	**duck**	SF English	鸭子	LF Mandarin
**animals**	**bear**	SF English	熊	LF Mandarin
**animals**	**horse**	SF English	马	LF Mandarin
**animals**	**bird**	SF English	鸟	SF Mandarin
**animals**	**dog**	SF English	狗	SF Mandarin
**body_parts**	**hair**	LF English	头发	SF Mandarin
**body_parts**	**knee**	LF English	膝盖	SF Mandarin
**body_parts**	**tongue**	LF English	舌头	SF Mandarin
**body_parts**	**chin**	SF English	下巴	LF Mandarin
**body_parts**	**ear**	SF English	耳朵	LF Mandarin
**body_parts**	**leg**	SF English	腿	LF Mandarin
**body_parts**	**hand**	SF English	手	SF Mandarin
**classifiers**	**a "person"**	-	位	SF Mandarin
**classifiers**	**strip**	-	条	SF Mandarin
**classifiers**	**a block(s)**	-	块	SF Mandarin
**clothing**	**shirt**	LF English	上衣	SF Mandarin
**clothing**	**vest**	LF English	背心	SF Mandarin
**clothing**	**zipper**	LF English	拉锁	SF Mandarin
**clothing**	**hat**	SF English	帽子	LF Mandarin
**clothing**	**necklace**	SF English	项链	LF Mandarin
**clothing**	**sock**	SF English	袜子	LF Mandarin
**clothing**	**shoe**	SF English	鞋 (子)	SF Mandarin
**connecting_words**	**because**	LF English	因为	SF Mandarin
**connecting_words**	**if**	SF English	如果，要是	LF Mandarin
**descriptive_words**	**Great Job! Well done!**	-	（真）棒!	SF Mandarin
**descriptive_words**	**good**	LF English	好	SF Mandarin
**descriptive_words**	**fragrant**	LF English	香	SF Mandarin
**descriptive_words**	**hot**	LF English	烫	SF Mandarin
**descriptive_words**	**pitiful**	LF English	可怜	SF Mandarin
**descriptive_words**	**enough**	LF English	够了	SF Mandarin

**descriptive_words**	**(be) careful**	LF English	小心	SF Mandarin
**descriptive_words**	**stuck**	LF English	卡了	SF Mandarin
**descriptive_words**	**two**	LF English	二	SF Mandarin
**descriptive_words**	**angry**	LF English-British	生气	SF Mandarin
**descriptive_words**	**gentle**	SF English	温柔	-
**descriptive_words**	**tiny**	SF English	非常小	-
**descriptive_words**	**hot**	SF English	烫	LF Mandarin
**descriptive_words**	**all gone**	SF English	没了	LF Mandarin
**descriptive_words**	**cold**	SF English	冷	LF Mandarin
**descriptive_words**	**fast**	SF English	快	LF Mandarin
**descriptive_words**	**happy**	SF English	高兴，快乐，开心	LF Mandarin
**descriptive_words**	**last**	SF English	最后	LF Mandarin
**descriptive_words**	**wet**	SF English	湿	LF Mandarin
**food_drink**	**bean sprout**	-	豆芽	SF Mandarin
**food_drink**	**water**	LF English	水	SF Mandarin
**food_drink**	**porridge**	LF English	粥	SF Mandarin
**food_drink**	**dumpling**	LF English	饺子	SF Mandarin
**food_drink**	**mushroom**	LF English	木耳	SF Mandarin
**food_drink**	**fruit**	LF English	水果	SF Mandarin
**food_drink**	**pear**	LF English	梨	SF Mandarin
**food_drink**	**(chili) pepper**	LF English	辣椒	SF Mandarin
**food_drink**	**applesauce**	SF English	苹果酱	-
**food_drink**	**milk**	SF English	牛奶	LF Mandarin
**food_drink**	**peas**	SF English	豌豆	LF Mandarin
**food_drink**	**coke**	SF English	可乐	LF Mandarin
**food_drink**	**cracker**	SF English	饼干	LF Mandarin
**food_drink**	**juice**	SF English	果汁	LF Mandarin
**food_drink**	**candy**	SF English	糖果	LF Mandarin (Taiwan)
**food_drink**	**meat**	SF English	肉	SF Mandarin
**furniture_rooms**	**coffee table**	-	茶几	SF Mandarin
**furniture_rooms**	**stove**	LF English	炉子/灶	SF Mandarin
**furniture_rooms**	**bench**	SF English	长椅	-
**furniture_rooms**	**oven**	SF English	烤箱	-
**furniture_rooms**	**bedroom**	SF English	卧室	LF Mandarin
**furniture_rooms**	**stairs**	SF English	楼梯	LF Mandarin
**furniture_rooms**	**bed**	SF English	床	SF Mandarin
**games_routines**	**(game) piece**	-	棋子	SF Mandarin
**games_routines**	**please**	LF English	请	SF Mandarin
**games_routines**	**shopping**	SF English	逛街	-

**games_routines**	**bye**	SF English	**再见/Byebye**	LF Mandarin
**games_routines**	**no**	SF English	**没，不，不是**	LF Mandarin
**games_routines**	**thank you**	SF English	**谢谢**	LF Mandarin
**games_routines**	**hi**	SF English	**你好**	SF Mandarin
**helping_verbs**	**could**	SF English	**能**	SF Mandarin
**helping_verbs**	**need/need to**	SF English	**要**	SF Mandarin
**helping_verbs**	**would**	SF English	**肯**	SF Mandarin
**household**	**bowl**	LF English	**碗**	SF Mandarin
**household**	**fan**	LF English	**电扇**	SF Mandarin
**household**	**basin / tub**	LF English	**盆**	SF Mandarin
**household**	**dustpan**	LF English	**簸箕**	SF Mandarin
**household**	**drawer**	LF English	**抽屉**	SF Mandarin
**household**	**tray**	SF English	**托盘**	-
**household**	**comb**	SF English	**梳子**	LF Mandarin
**household**	**mop**	SF English	**拖把**	LF Mandarin
**household**	**plate**	SF English	**盘子**	LF Mandarin
**household**	**towel**	SF English	**毛巾**	LF Mandarin
**household**	**trash**	SF English	**垃圾**	LF Mandarin
**household**	**broom**	SF English	**笤扫，扫帚**	SF Mandarin & -
**outside**	**flower**	LF English	**花**	SF Mandarin
**outside**	**cloud**	LF English	**云**	SF Mandarin
**outside**	**stick**	LF English	**棍子**	SF Mandarin
**outside**	**branch**	LF English	**树枝**	SF Mandarin
**outside**	**rain**	SF English	**(下)雨**	LF Mandarin
**outside**	**flag**	SF English	**旗（子）**	LF Mandarin
**outside**	**sky**	SF English	**天空**	LF Mandarin
**outside**	**star**	SF English	**星星**	LF Mandarin
**outside**	**swing**	SF English	**秋千**	LF Mandarin
**particles**	**of course / duh (or another word your family uses to indicate something is obvious)**	-	**嘛**	SF Mandarin
**particles**	**okay (or another word your family uses to indicate acknowledgment)**	-	**噢 ,哦, 噢, 喔**	SF Mandarin
**people**	**baby**	LF English	**宝宝**	SF Mandarin

**people**	**brother**	LF English	哥哥/弟弟	SF Mandarin
**people**	**aunt**	LF English	阿姨	SF Mandarin
**people**	**teacher**	LF English	老师	SF Mandarin
**people**	**mommy**	SF English	妈妈	LF Mandarin
**people**	**friend**	SF English	朋友	LF Mandarin
**people**	**person**	SF English	人	LF Mandarin
**places**	**-**	-	中国	SF Mandarin
**places**	**zoo**	LF English	动物园	SF Mandarin
**places**	**kindergarten**	LF English	幼儿园/托儿所	SF Mandarin
**places**	**party**	SF English	聚会 / 派对	-
**places**	**school**	SF English	学校	LF Mandarin
**prepositions_locations**	**towards**	LF English	朝	SF Mandarin
**prepositions_locations**	**down**	SF English	下去	-
**prepositions_locations**	**beside**	SF English	旁边	SF Mandarin
**prepositions_locations**	**under**	SF English	底下	SF Mandarin
**pronouns**	**myself, self**	-	自己	SF Mandarin
**pronouns**	**other people**	-	人家	SF Mandarin
**pronouns**	**that**	LF English	那个	SF Mandarin
**pronouns**	**them**	SF English	他们/她们/它们	-
**pronouns**	**us**	SF English	我们	-
**pronouns**	**this**	SF English	这个	LF Mandarin
**pronouns**	**our**	SF English	我们的	SF Mandarin
**quantifiers_articles**	**some**	-	些	SF Mandarin
**quantifiers_articles**	**thing**	LF English	东西	SF Mandarin
**quantifiers_articles**	**much**	SF English	好多，很多	LF Mandarin (Taiwan)
**quantifiers_articles**	**all**	SF English	全部	SF Mandarin
**question_words**	**which**	LF English	哪个	SF Mandarin
**question_words**	**how much / how many**	LF English	多少	SF Mandarin
**question_words**	**where**	SF English	哪里，在哪儿，哪儿呢	SF Mandarin
**sounds**	**uh oh**	SF English	呃哦	-
**sounds**	**woof woof (dog sound)**	SF English	旺旺（狗叫）	LF Mandarin
**sounds**	**baa baa (sheep sound)**	SF English	咩咩（羊叫声）	LF Mandarin
**sounds**	**ouch**	SF English	哎哟	SF Mandarin
**sounds**	**meow (cat sound)**	SF English	喵（猫叫）	SF Mandarin

**time_words**	**today**	LF English	今天	SF Mandarin
**time_words**	**already**	LF English	已经	SF Mandarin
**time_words**	**before**	LF English	以前	SF Mandarin
**time_words**	**tonight**	SF English	今晚	-
**time_words**	**day**	SF English	天	LF Mandarin
**time_words**	**after**	SF English	以后, 之后	LF Mandarin & -
**toys**	**book**	SF English	书	LF Mandarin
**toys**	**game**	SF English	游戏	LF Mandarin
**toys**	**ball**	SF English	球	SF Mandarin
**vehicles**	**truck**	LF English	卡车	SF Mandarin
**vehicles**	**airplane**	SF English	飞机	LF Mandarin
**vehicles**	**boat**	SF English	船	LF Mandarin
**vehicles**	**car**	SF English	车	SF Mandarin
